# High-Contrast Lumbar Spinal Bone Imaging Using a 3D Slab-Selective UTE Sequence

**DOI:** 10.3389/fendo.2021.800398

**Published:** 2022-01-07

**Authors:** Amir Masoud Afsahi, Alecio F. Lombardi, Zhao Wei, Michael Carl, Jiyo Athertya, Koichi Masuda, Mark Wallace, Roland R. Lee, Ya-Jun Ma

**Affiliations:** ^1^ Department of Radiology, University of California San Diego, San Diego, CA, United States; ^2^ Research Service, Veterans Affairs San Diego Healthcare System, San Diego, CA, United States; ^3^ GE Healthcare, San Diego, CA, United States; ^4^ Department of Orthopedic Surgery, University of California San Diego, San Diego, CA, United States; ^5^ Department of Anesthesiology, University of California San Diego, San Diego, CA, United States

**Keywords:** UTE MRI, bone imaging, lumbar spine, slab selective, CT-like contrast, ZTE MRI

## Abstract

Ultra-short echo time (UTE) MRI with post-processing is a promising technique in bone imaging that produces a similar contrast to computed tomography (CT). Here, we propose a 3D slab-selective ultrashort echo time (UTE) sequence together with image post-processing to image bone structures in the lumbar spine. We also explore the intermodality agreement between the UTE and CT images. The lumbar spines of two healthy volunteers were imaged with 3D UTE using five different resolutions to determine the best imaging protocol. Then, four patients with low back pain were imaged with both the 3D UTE sequence and CT to investigate agreement between the imaging methods. Two other patients with low back pain were then imaged with the 3D UTE sequence and clinical conventional T_1_-weighted and T_2_-weighted fast spin-echo (FSE) MRI sequences for qualitative comparison. The 3D UTE sequence together with post-processing showed high contrast images of bone and high intermodality agreement with CT images. In conclusion, post-processed slab-selective UTE imaging is a feasible approach for highlighting bone structures in the lumbar spine and demonstrates significant anatomical correlation with CT images.

## Introduction

Spinal disorders are a major medical, social, and economic issue due to their high prevalence and increasing incidence, especially among the elderly (1). Computed tomography (CT) offers both high-resolution and high-contrast imaging of bone, but due to the modality’s involvement of ionizing radiation exposure, it is not recommended for children or patients who require frequent examinations. Most recently, zero echo time (ZTE) combined with data post-processing (2) has been applied for high contrast bone imaging of the head (3), shoulder (4), cervical spine (5), and hip (6). ZTE imaging uses a non-selective radiofrequency (RF) pulse (i.e., a short rectangular pulse) for signal excitation in which all body parts are excited simultaneously. In addition, ZTE uses the shortest echo time achievable in the MR system and some coils require a relatively long ring-down time after excitation (between 10 and 200 µs) (7, 8). Data acquired during this ring-down period may lead to spatial signal inhomogeneity.

Ultrashort echo time (UTE) is an MR technique that also enables sufficiently fast data acquisition to detect signals from bone (9, 10). UTE applies slab-selectivity using a half, soft RF pulse for signal excitation during a slice-selective gradient that reduces artifacts related to respiratory motion. That way, UTE can selectively excite the spine without simultaneous excitation of the adjacent organs. In addition, echo time is more easily adjusted in the UTE sequence minimizing artifacts induced by the coil ring-down. Moreover, UTE MRI allows for visualization of structures in the spine that are not directly seen with conventional MRI due to their rapid signal decay, including pars interarticularis, longitudinal ligaments, annulus fibrosus, and the cartilaginous endplate (11, 12). For example, it has been shown that the diagnostic confidence for spondylolysis in cadaveric spines is higher when using UTE MRI than either conventional gradient-echo or short tau inversion recovery (STIR) sequences (13). Another potential application—and advantage—of UTE sequences over conventional MRI may be the quantitative assessment of enthesis in patients with inflammatory arthropathies (14, 15).

In this study, we investigated the performance of a 3D slab-selective UTE sequence together with signal post-processing to image bone structures in the lumbar spine in comparison with CT. We hypothesized that the post-processed slab-selective UTE images could show bone structures in the lumbar spine with high resolution and a high intermodality agreement with CT.

## Methods

### MR Acquisition and *In Vivo* Study

**Figure 1:**
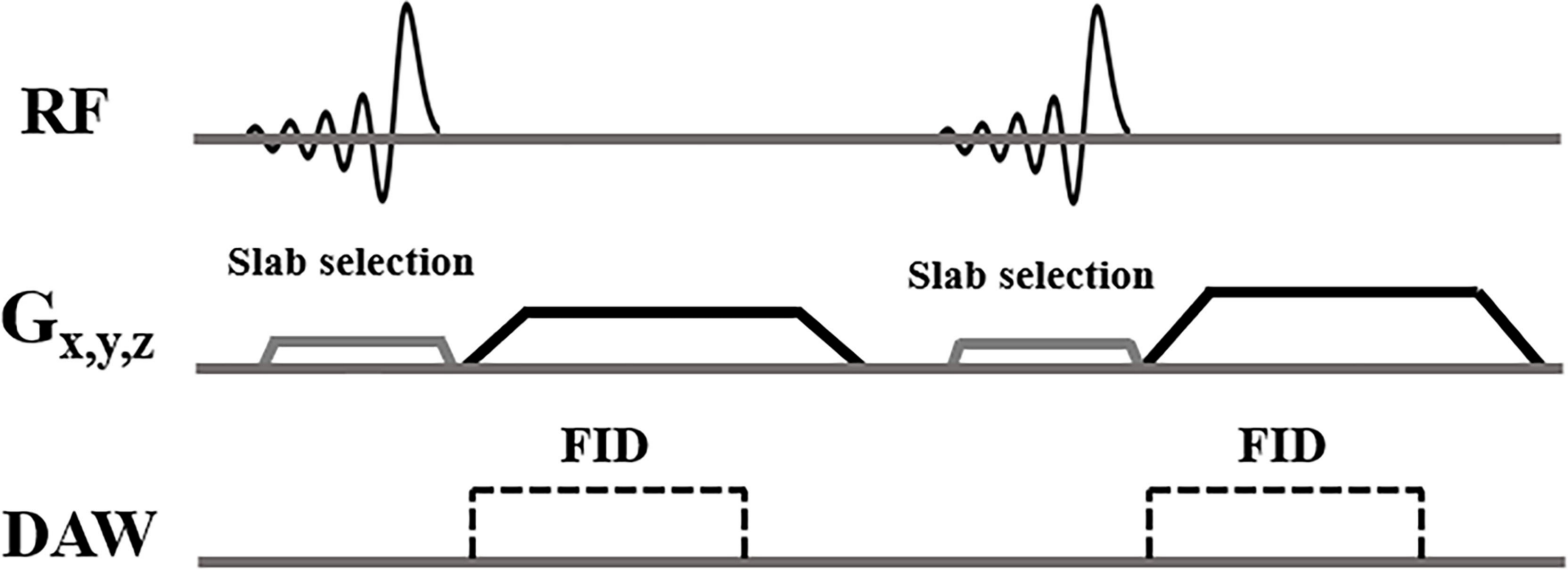
3D slab-selective UTE sequences. The 3D UTE sequence enables slab selection by using a half, soft pulse for excitation together with a slice-selective gradient. After excitation, the spatial encoding gradient is turned on and simultaneous data acquisition begins.

Informed consent was obtained from all subjects following the guidelines of the local Institutional Review Board. All sequences were implemented on a 3T GE MR750 scanner (GE Healthcare Technologies, Milwaukee, WI) and a standard spine coil was used for signal reception. **Figure 1** shows the features of the 3D slab-selective UTE sequence. The UTE sequence enables slab selection by using a slice-selective short, half pulse (Shinnar-Le Roux (SLR) design, duration = 628 µs, and bandwidth = 15 kHz) with a variable-rate selective excitation (VERSE) design for excitation (16). After excitation, the spatial-encoding gradient is turned on and simultaneous data acquisition begins. As a result, the UTE data close to the k-space center are acquired on the gradient ramp.

All UTE scans were performed in the coronal plane to exclude soft tissues in the abdomen (i.e., the major structures involved in respiratory motion artifacts). A 5-second calibration scan was performed to correct the coil sensitivity inhomogeneity. To optimize the UTE sequence parameters and determine the best resolution and contrast, five scans with different isotropic resolutions (i.e., 0.9, 1.0, 1.2, 1.6, and 2 mm^3^) were performed on the two abovementioned healthy volunteers (detailed sequence parameters in **Table 1**). Two experienced radiologists (A.F.L. and E.Y.C.) reviewed the images of different resolutions and determined which UTE imaging protocol performed best qualitatively. The final UTE protocol (i.e., with a resolution of 1.2 mm^3^), (see the *Results* section) was then tested by scanning five healthy volunteers (28 to 48 years old, three males, two females). Oversampling in UTE imaging is beneficial for improving signal-to-noise ratio (SNR) and for reducing aliasing artifacts. Therefore, we oversampled the UTE acquisitions to improve the image quality for a scan that ran about 9 min. We also performed UTE MRI with and without RF pulse slab-selectivity (with the same sequence parameters) in the lumbar spine of a 40-year-old male healthy volunteer to compare performance in bone imaging.

**Table 1:** UTE sequence parameters for different resolutions.

Seq Scan	FOV (cm^3^)	Resolution (mm^3^)	TR/TE (ms)	FA	BW (kHz)	Oversampling Factor	Average	Scan Time (min)
**#1**	32x32x7.2	0.9x0.9x0.9	2.4/0.028	1°	125	1	2	9.6
**#2**	32x32x8.0	1.0x1.0x1.0	2.2/0.028	1°	125	1.1	2	9.3
**#3**	32x32x9.6	1.2x1.2x1.2	2.0/0.028	1°	125	1.5	2	9
**#4**	32x32x9.6	1.6x1.6x1.6	1.7/0.028	1°	125	2.2	2	6.5
**#5**	32x32x12	2.0x2.0x2.0	1.6/0.028	1°	125	2.2	2	4.8

FOV, field-of-view; TR, repetition time; TE, echo time; FA, flip angle; BW, bandwidth.

Next, four patients (46 to 75 years old, all males) with low back pain were recruited and underwent UTE imaging for comparison against their most recent CT images.

Finally, two other patients (42 years old and 35 years old, both males) with low back pain were recruited and underwent both UTE imaging and conventional MR sequences [T_1_-weighted fast spin-echo (T_1w_-FSE) and T_2_-weighted FSE (T_2_w-FSE)] for comparison.

### Bone Imaging Post-Processing

The standardized post-processing procedure available in the workstation of the GE scanner was automatically completed for the acquired UTE images to render images with a ‘‘CT-like’’ contrast between bone and soft tissue (3, 17). The procedure includes an N4ITK bias correction algorithm (18), which was first applied to correct for intensity irregularities in the acquired UTE images due to the transmit coil profile inhomogeneity. Then, the contrasts of these bias-corrected images were logarithmically transformed and inverted. The process required approximately 1 to 3 minutes.

### Comparison Between Non-Selective and Slab-Selective UTE Acquisitions

UTE imaging of the lumbar spine with non-selective and slab-selective RF pulses were performed in a healthy volunteer to compare focus measure and image blurring. A quantitative measure of focus was performed by placing a Laplacian filter on the image to detect edges, then performing a calculation of the variance of the filtered image (19). Convolution of a 3x3 Laplacian kernel was applied to the UTE images to detect tissue edges, with a higher variance measurement implying greater image sharpness/less blurring. OpenCV python library was used to perform the image convolution and focus measurement (20).

### Automatic Image Registration for Intermodality Agreement Evaluation

To demonstrate the morphological similarities of images between our proposed UTE sequence and CT, a 3D volumetric registration framework using the Insight Toolkit (ITK) (21) was implemented in the four patients submitted to both UTE and CT images. First, the 3D volumetric images were pre-processed to highlight the targeting bone and minimize soft tissue contrast. Then, we performed rigid registration with a centered transform initializer which provides the initial center of rotation and translation. This step aligns each respective geometric center of the two volumes. The rigid registration used the mutual information as the similarity metric with gradient descent optimization. The number of iterations was 100 with a learning rate of 1.0. The sampling strategy was set to random, and the interpolator type set to linear.

### Statistical Analysis

In addition to the quantitative image registration framework, one fellowship-trained musculoskeletal radiologist reviewed the images of the four patients submitted to both UTE and CT images, for intermodality agreement comparison. Measurements of vertebral body height and anteroposterior length using reconstructed sagittal CT and UTE MR images were performed in 20 vertebral bodies. A mid-sagittal plane was chosen for the measurements as it showed all the vertebral bodies simultaneously, in contrast with the coronal plane in which the lumbar lordosis prevented the simultaneous visualization of all vertebral bodies. The Shapiro-Wilk test was performed to test for normality of data distribution and a nonparametric statistics test was used to compare mean values between both image methods. Bias and limit-of-agreement (LoA) values (1.96 x standard deviation of difference) were calculated using the Bland and Altman method (22) and analyses were performed using R v.4.0.2 (R Core Team, 2014) and RStudio v.1.3.1073 (RStudio Core Team, 2020) (23, 24). *P* <.05 was considered as statistically significant.

## Results

**Figure 2:**
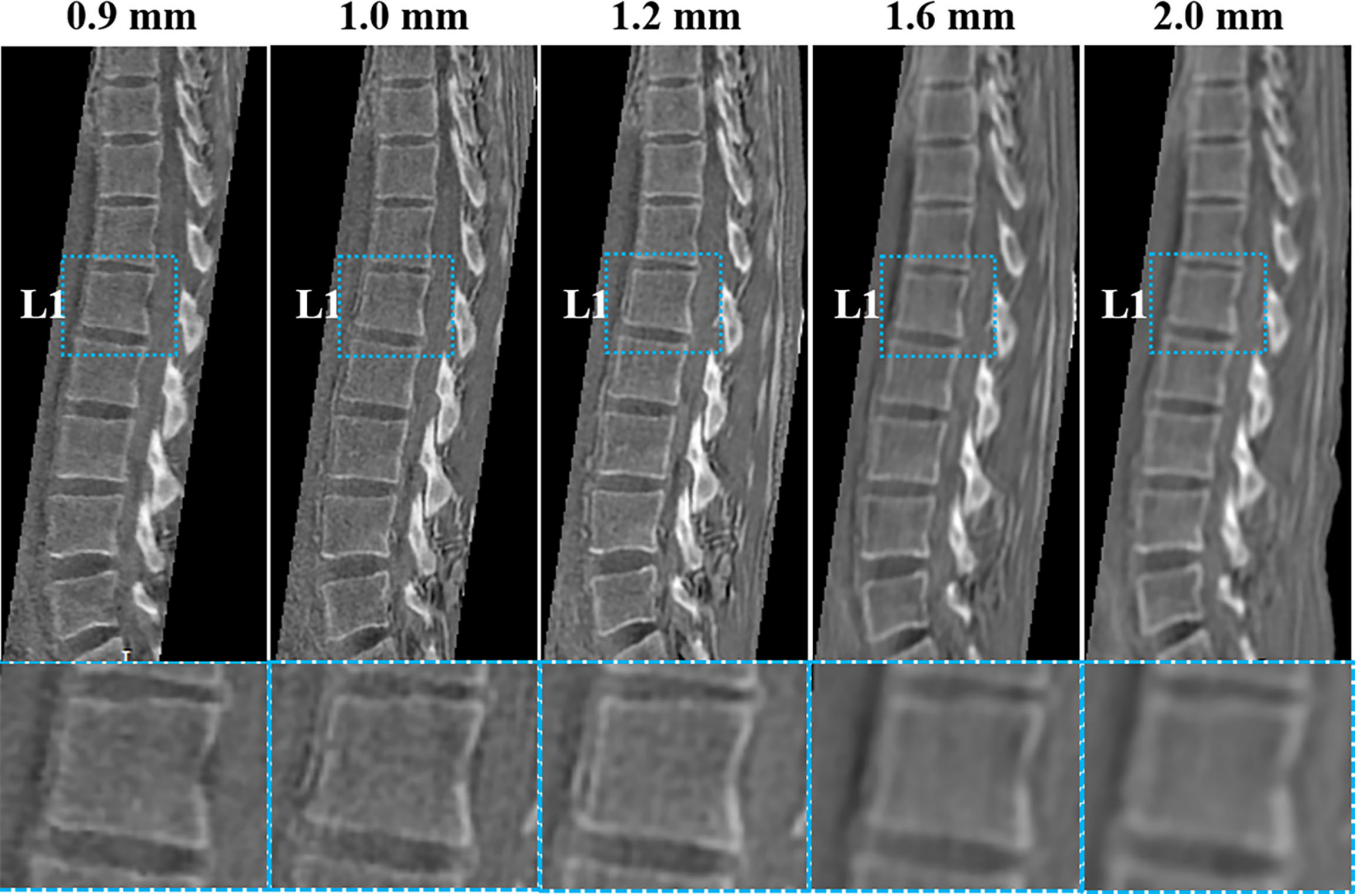
UTE images with five different resolutions (0.9, 1.0, 1.2, 1.6, and 2 mm^3^) of a healthy volunteer (29-year-old female). The corresponding UTE zoomed-in images of L1 (blue boxes in second row) are shown for better comparison. The resolution with a voxel size of 1.2 mm^3^ shows a good balance between image SNR and bone structure visualization.

**Figure 2** shows the five 3D UTE scans with different resolutions from the same healthy volunteer. The SNR was lower when the resolution was higher, but when the resolution was too low, the bone structures were not well-demonstrated. The resolution with a voxel size of 1.2 mm^3^ showed a good balance between image SNR and resolution of bone structures, as qualitatively verified by two radiologists.

As seen in **Supplementary Figure 1**, the slab-selective UTE acquisition shows the boundaries of bone more clearly than the non-slab-selective acquisition. The focus measure of the slab-selective UTE image shows a value about 2.5x higher value than that of the non-slab-selective image in the lumbar vertebral bone region, demonstrating that the slab-selective UTE acquisition produces better bone sharpness than the non-slab-selective acquisition due to its greater capability to alleviate the interference of respiratory motion artifacts. On the other hand, no apparent motion artifacts were observed in the non-slab-selective UTE images. This is because the non-Cartesian acquisition is inherently less sensitive to motion artifacts than a Cartesian acquisition and the artifacts are shown as image blurriness as can be seen in **Supplementary Figure 1**. Moreover, because of the motion average effect, the oversampling strategy also helps reduce the artifacts included by the periodical respiratory motion. For registration, all the images were pre-processed with modified contrast and reduced resolution in order to facilitate a faster and better registration between the UTE and CT images and, as a result, the image quality in **Supplementary Figure 1** was not only lower than that of the images shown in the remaining figures but also inadequate for diagnosis.

**Figure 3:**
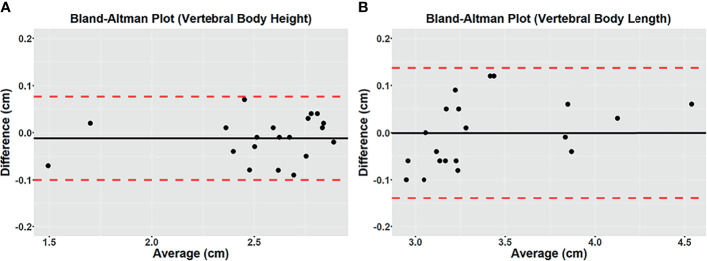
Bland-Altman plots of differences between 3D CT and 3D UTE MRI for vertebral body height **(A)** and vertebral body length **(B)** measured on the mid-sagittal plane from the *in vivo* study. A solid line represents the mean of all differences (bias), while a dashed line indicates the 95% interval confidence of agreement. All measurements were within the 95% confidence interval limits.

The high intermodality agreement was also confirmed using the rigid registration framework. A representative example showing the correlation between specific points in one normal and one fractured vertebral body on both imaging methods is shown in **Supplementary Figure 2**. Using vertebral body height and anteroposterior vertebral body length as comparison measures among the four patients with back pain, we found high intermodality agreement. The summary of the vertebral body height and anteroposterior vertebral body length measurements can be found in **Table 2**. Similar values with no statistical differences were found in these measurements for the CT and UTE images (*P* >.05). **Figure 3** shows the corresponding Bland-Altman plots. The LoAs in our data include more than 95% of differences between the two measurement methods (*P* <.01), reinforcing the similarities that exist between them.

**Table 2:** Summary of the mean vertebral bodies’ height and length of patients measured on CT and UTE MRI.

		VB Height (mm)		VB Length (mm)		Wilcoxon
		CT	UTE	CT	UTE	
**Patient 1**	L1	2.83	2.79	3.88	3.82	p >.05
	L2	2.85	2.83	4.14	4.11	
	L3	1.71	1.69	4.57	4.51	
	L4	2.78	2.75	3.83	3.84	
	L5	2.8	2.76	3.5	3.38	
**Patient 2**	L1	2.67	2.68	3	3.1	p >.05
	L2	2.65	2.74	3.11	3.17	
	L3	2.73	2.78	3.1	3.14	
	L4	2.88	2.9	2.93	2.99	
	L5	2.84	2.83	2.9	3.00	
**Patient 3**	L1	2.37	2.36	3.29	3.28	p >.05
	L2	2.49	2.52	3.2	3.28	
	L3	2.44	2.52	3.2	3.26	
	L4	2.62	2.63	3.14	3.2	
	L5	2.6	2.59	3.27	3.18	
**Patient 4**	L1	2.49	2.42	3.06	3.06	p >.05
	L2	2.51	2.52	3.2	3.15	
	L3	2.58	2.66	3.27	3.22	
	L4	14.6	15.3	3.85	3.89	
	L5	23.8	24.2	34.8	33.6	

VB, Vertebral body; Wilcoxon, Wilcoxon rank sum test.

**Figure 4:**
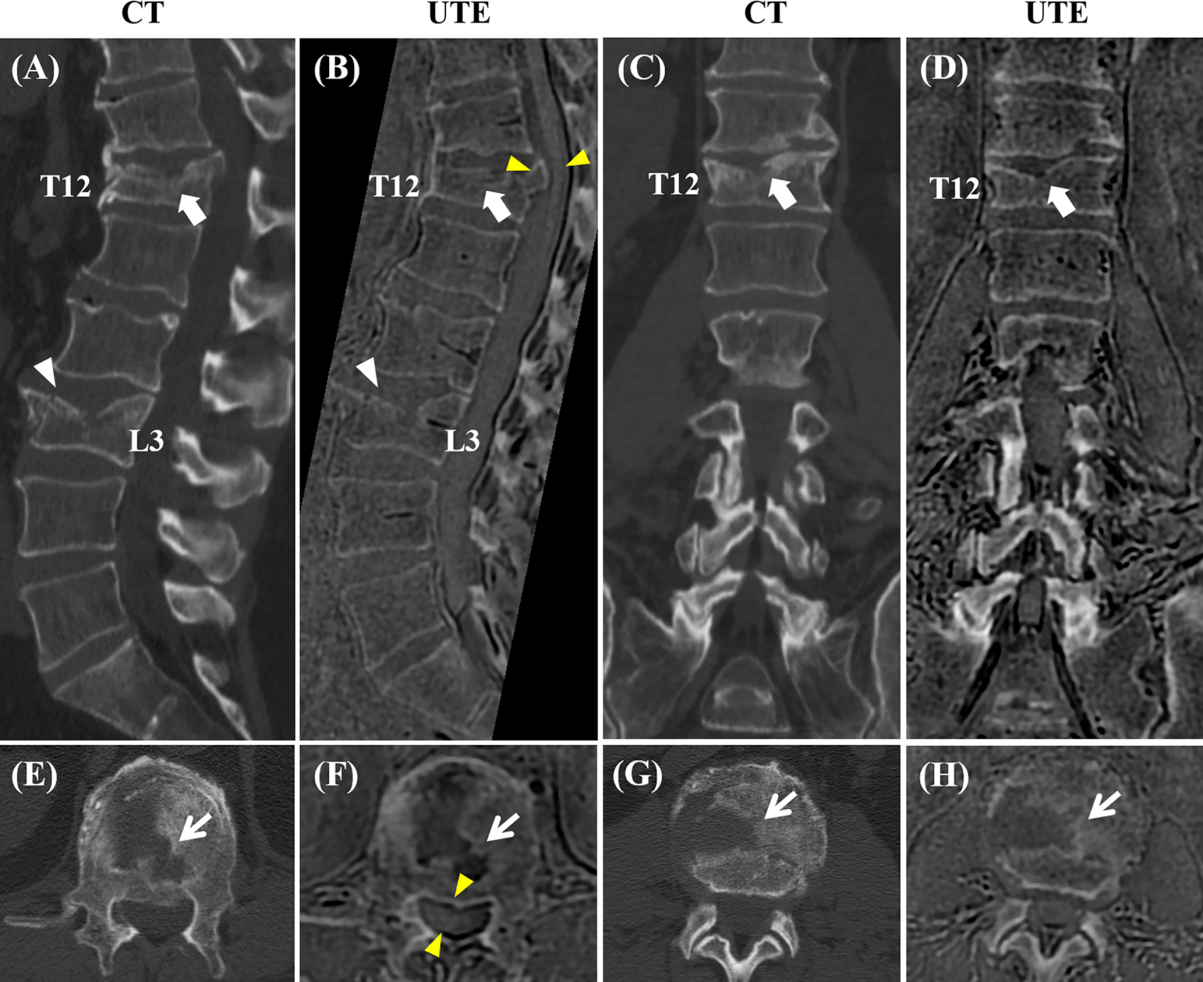
Correlation between CT and the 3D slab-selective UTE MRI sequence in a 72-year-old male with low back pain (T11-L5). Compression fractures are seen in T12 and L3 on the CT image (arrow and arrowhead in **(A)**, respectively), showing excellent anatomic correlation with the 3D slab-selective UTE sequence (arrow and arrowhead in **(B)**, respectively). Coronal **(C, D)** and axial **(E–H)** images also showed excellent anatomic correlation of the fractures between the CT scan and the 3D slab-selective UTE sequence (arrows in **(E–H)**. Note the retropulsion of a bone fragment in T12 narrowing the spinal canal as well as its proximity to the spinal cord which can be seen on the UTE sequence (yellow arrowheads in **(B, F)**.

**Figure 4** shows one example of both modalities being applied to the lumbar spine of a patient with low back pain. The boundaries of the vertebral bodies have similar contrast and resolution, and the bone fractures can be visualized on both CT and UTE MRI in all three planes.

**Figure 5:**
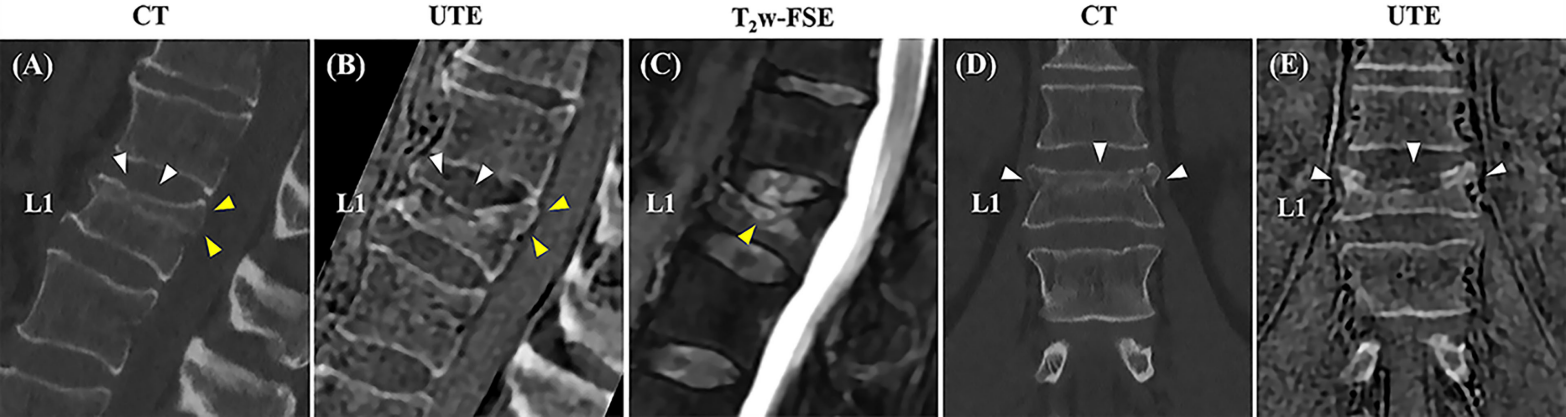
Correlation between sagittal and coronal CT images and the 3D slab-selective UTE MRI sequence obtained three months apart in a 55-year-old male with low back pain. A compression fracture is seen in L1 on both the CT images (white arrowheads in **A**, **D**) and clinical MR image **(C)** associated with retropulsion of the posterior vertebral body cortex (yellow arrowheads in **A**). The 3D slab-selective UTE sequence shows the progression of the fracture with further loss of vertebral body height and fragmentation of the upper endplate (white arrowheads in **B**, **E**) in comparison to CT images. Note also the retropulsion of the posterior vertebral body cortex (yellow arrowheads in **B**).

**Figure 5** shows a comparison between CT and UTE MRI from a 55-year-old patient with low back pain. The MR images were acquired four months after the CT images. On the MRI, the fracture in L1 shows reduced vertebral body height and superior endplate fragmentation compared to the previous CT. In this case, MRI allowed for the evaluation of bone marrow edema using the T_2_-weighted sequence and suggested acute or subacute fracture, information not available when using only CT images.

**Figure 6:**
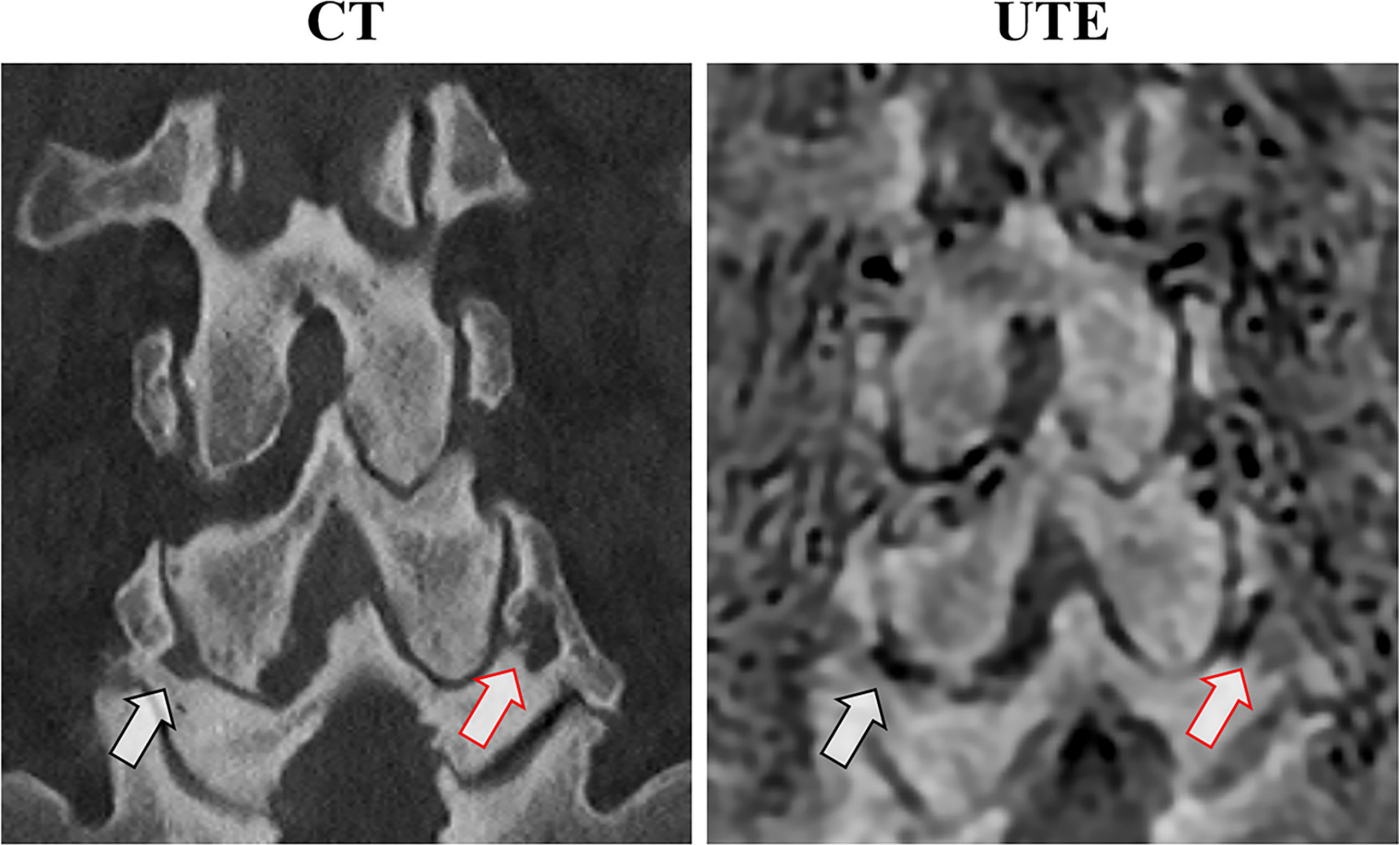
Anatomical correlation between coronal CT and the 3D slab-selective UTE MRI sequence in a 75-year-old male with low back pain. Note the bilateral facet joint osteoarthritis with subchondral irregularities and cysts (arrows).

**Figure 6** shows a comparison between CT and 3D UTE in another patient with low back pain. Facet joint osteoarthritis, characterized by joint space narrowing and subchondral bone sclerosis, as well as subchondral bone cysts can be seen in both methods with good morphological correlation.

**Figure 7:**
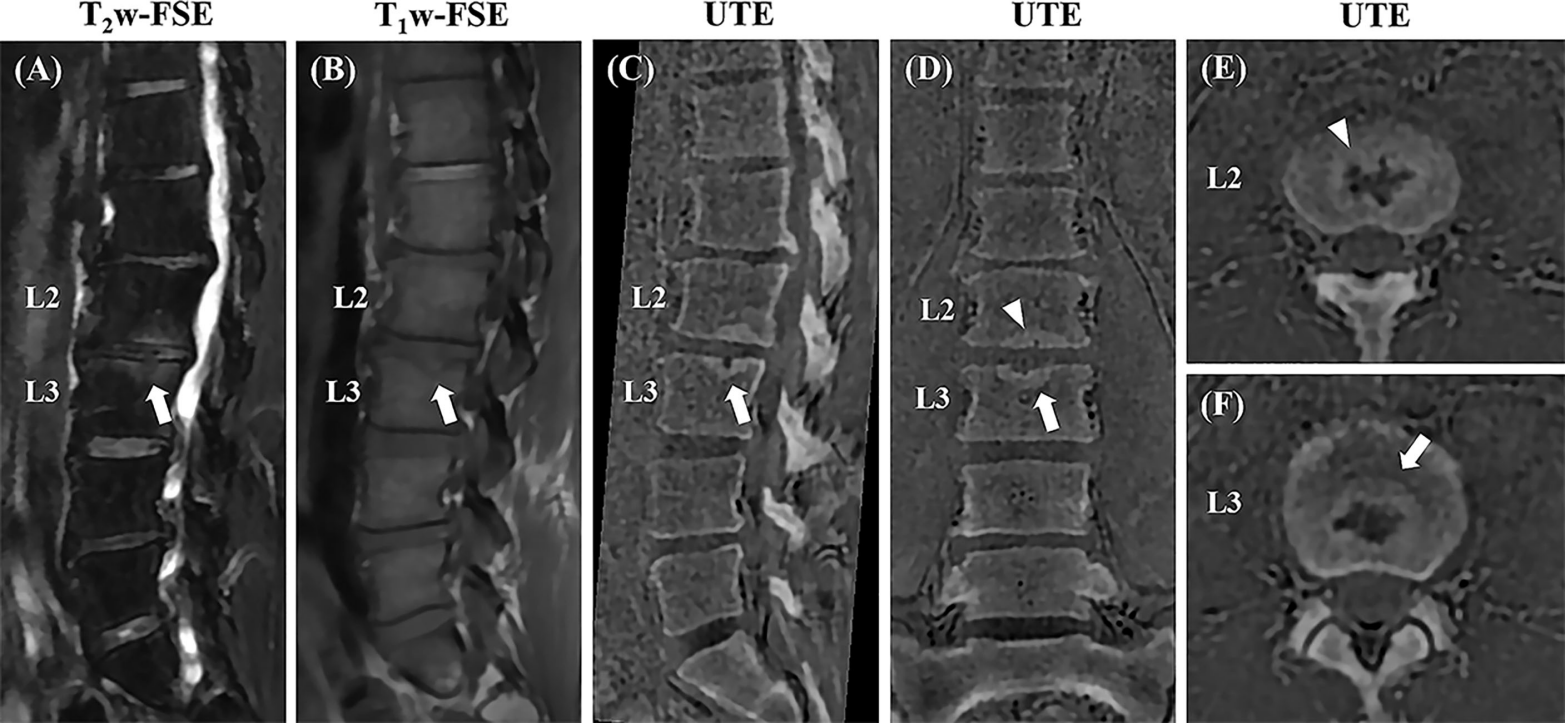
Sagittal T_2_w-FS and T_1_w-FSE images **(A, B)** and sagittal **(C)**, coronal **(D)**, and axial **(E, F)** 3D slab-selective UTE images from a 42-year-old male patient with low back pain (T12-L5). Note the Schmorl’s node in the superior endplate of L3 (arrows in **(A, B)** which demonstrates excellent anatomic correlation with the 3D slab-selective UTE sequence on the sagittal **(C)**, coronal **(D)**, and axial **(E, F)** images (arrows). Another Schmorl’s node can be seen in the inferior endplate of L2 [arrowheads in **(D, E)**].

Schmorl’s nodes (**Figure 7**) showed more conspicuity on UTE MRI, compared to conventional MRI sequences. In addition, chronic pars interarticularis fractures that may sometimes be hard to diagnose using T_1_-weighted or T_2_-weighted sequences were easily characterized on the 3D UTE sequence as seen in **Supplementary Figure 3**.

## Discussion

In this study we showed that a 3D slab-selective UTE sequence together with post-processing is feasible for bone imaging of the lumbar spine, displaying both high contrast and excellent anatomical correlation with CT images. This supports the argument for the potential implementation of the 3D UTE sequence into the clinical workflow.

The UTE sequence uses a soft RF pulse for signal excitation, offering the possibility of slab selectivity as a way to limit the 3D FOV and potentially reduce its sensitivity to respiratory motion artifacts (25). This is especially advantageous in the clinical evaluation of the dorsal and lumbar spine. Additionally, more efficient k-space trajectories such as spirals (26), twisted (27), or cones (28) are possible in UTE imaging because the UTE sequence readout gradient is applied after the RF pulse, ultimately facilitating reduction of SNR issues and scan time. These prolonged readout gradients together with the inherent ramp sampling in UTE sequence add more T_2_* contrast between bone and soft tissues and facilitate further highlighting of the bone in post-processed images.

The post-processed UTE images showed bone structures with high resolution and high SNR. The vertebral body contours were well-defined, especially in the anterior portion of the spine which is in contact with abdominal organs and, therefore, more subject to respiratory motion artifacts (see **Figures 4** and **5**). There was high agreement between the UTE and CT images, showing excellent anatomical correlation between both methods as demonstrated by the image registration framework and the Bland-Altman analysis. Therefore, the UTE-based bone imaging may be useful for longitudinal studies or studies involving children given that UTE poses no threat of ionizing radiation exposure for the patient. Furthermore, lesions such as the Schmorl’s nodes and other compressive fractures were also clearly seen on the UTE images, with clinical MRI used as the reference standard.

The UTE image shows some differences with the corresponding CT images as can be seen in **Figure 7**. This is probably because the slice thickness and patient positioning (non-rigid motion) were different between UTE and CT scans. It is technically challenging to get a perfect registration for non-rigid motion, especially for the small structures. A more advanced registration technique will be used to improve the co-registration in our future study (29).

There are more complicated structures in the UTE images than in the corresponding CT images. This is because UTE MRI can detect signals from all kinds of soft tissues with high signal intensities. The soft tissue boundaries may also be highlighted due to the fast T_2_* decay (induced by the susceptibility differences between tissues) in these regions. Moreover, the fatty tissue may create more contrast due to the chemical shift artifacts in non-Cartesian UTE acquisition. ZTE imaging may be preferable in terms of reducing this off resonance-induced contrast since it has a shorter effective echo time than UTE. This will be investigated in a future UTE and ZTE comparison study.

Degenerative alterations and compressive fractures can be easily detected by clinical MRI. We propose that the UTE MRI technique be used as a complementary modality to improve MRI-only cortical and trabecular bone assessment, circumventing CT’s unnecessary ionizing radiation exposure. This is particularly appealing in the cases of young patients or patients who would need to undergo repeated examinations. However, in some anatomical regions, it may be difficult to determine whether there is indeed a fracture present on clinical MRI, such as was the case with our patient who had a pars interarticularis fracture. Other spinal diseases such as spinal tumors and infections will be investigated in our future studies. It is well recognized that the clinical MRI ability to differentiate acute or subacute from chronic vertebral body fractures (e.g., the patient represented in **Figure 5**) is a clear advantage over CT, especially when no previous exams are available for comparison and, as a result, UTE combined with clinical MRI may provide much more useful information for diagnosis than CT alone in clinical practice.

UTE MRI has been very useful to image the cartilaginous endplate (CEP). We have recently developed a new inversion recovery-prepared and fat-saturated UTE (IR-FS-UTE) sequence to highlight the CEP signal (30). However, in this study, a proton density-weighted UTE sequence (flip angle = 1°) was used for bone imaging. Even though the CEP has a short T_2_/T_2_*, its proton density is much higher than that of bone. Thus, the signal intensity of CEP is actually similar to that of other long T_2_ soft tissues and, as a result, we don’t expect that CEP signal would affect vertebral bone imaging. In addition, we would suggest using a highly T_1_-weighted UTE sequence (e.g., an increased excitation flip angle (e.g., 15°) in the UTE sequence) to image CEP as the CEP has a much shorter T_1_ than the nucleus pulposus (30).

This study has several limitations. First, a small number of subjects were used in this technical feasibility study. More patients will be recruited for a more comprehensive comparison of UTE MRI with CT and clinical MRI in a future study. Second, the 3D slab-selective UTE MRI can be reconstructed in three orthogonal planes; however, it is not yet feasible to complete 3D rendering at this stage. Technical developments to further improve the bone contrast for 3D rendering are under development and will be demonstrated in future studies. Third, CT is better at showing certain bone structures than UTE, such as fractured bones with soft tissue contamination. In such a case, the bone contrast in the UTE image is low due to the partial volume effect. UTE imaging with a higher resolution may solve this problem, but the scan time would significantly increase. Advanced fast imaging techniques, such as compressed sensing (31) and deep learning (32), could potentially be used to generate high resolution UTE images without compromising scan time.

## Conclusion

In conclusion, the proposed 3D slab-selective UTE sequence together with image post-processing is capable of imaging spinal bone with high resolution with high intermodality agreement with CT and is promising as a clinical technique in spinal bone imaging.

## Data Availability Statement

The original contributions presented in the study are included in the article/**Supplementary Material**. Further inquiries can be directed to the corresponding author.

## Ethics Statement

The studies involving human participants were reviewed and approved by IRB University of California San Diego. The patients/participants provided their written informed consent to participate in this study.

## Author Contributions

Y-JM contributed to conception and design of the study. AA, AL, ZW, MC, and JA organized the database. AL and JA performed the statistical analysis. AA, AL, and Y-JM wrote the first draft of the manuscript. JA, KM, MW, and RL wrote sections of the manuscript. All authors contributed to manuscript revision, read, and approved the submitted version.

## Funding

The authors acknowledge grant support from NIH (R21AR075851). The authors declare that this study received funding from GE Healthcare and that this funder was not involved in the study design, collection, analysis, interpretation of data, the writing of this article or the decision to submit it for publication.

## Conflict of Interest

MC was employed by the company GE Healthcare.

The remaining authors declare that the research was conducted in the absence of any commercial or financial relationships that could be construed as a potential conflict of interest.

## Publisher’s Note

All claims expressed in this article are solely those of the authors and do not necessarily represent those of their affiliated organizations, or those of the publisher, the editors and the reviewers. Any product that may be evaluated in this article, or claim that may be made by its manufacturer, is not guaranteed or endorsed by the publisher.

## Supplementary Material

Supplementary Figure 1 Coronal **(A–D)** and sagittal **(E–H)** UTE images with slab-selective and non-slab-selective RF pulses for signal excitation. Greater bone sharpness was observed in the slab-selective UTE images **(A, E)** than the corresponding non-slab-selective images **(B, F)**. The corresponding focus measure for the Laplacian filtered slab-selective UTE images **(C, G)** shows about 2.5x higher value than that of the non-slab-selective images **(D, H)**.

Supplementary Figure 2 Volumetric automatic rigid registration between CT and 3D UTE MRI on a normal vertebral body **(A, C)** and a fractured vertebral body **(B, D)**. A centered initialization was followed by rigid registration. The red crosses represent corresponding points automatically detected by the registration algorithm. The yellow dashed lines (fracture) and orange dashed circles (vessel) have been drawn to highlight regions for visual comparison.

Supplementary Figure 3 Sagittal T_2_w-FS and T_1_w-FSE images **(A, B)** and sagittal **(C)**, axial **(D)**, and coronal **(E)** 3D slab-selective UTE images from a 35-year-old male patient with low back pain (T12-L5). A spondylolysis of the pars interarticularis of L5 with anterior spondylolisthesis of L5 is better distinguished on the 3D slab-selective UTE MR sequence [arrows in **(A–C)**]. An axial oblique reconstructed image of the UTE MR sequence shows the bilateral fractures of the pars interarticularis in detail [arrows in **(D)**]. The coronal reconstructed image of the 3D slab-selective UTE MR sequence shows the corresponding expected location of the fractures [arrowheads in **(E)**] as well as a Schmorl’s node in the superior endplate of S1 [arrow in **(E)**].

## Abbreviations

UTE, Ultrashort Echo Time; ZTE, Zero Echo Time; CT, Computed Tomography; FSE, Fast Spin-Echo; PD, Proton Density; TE, Echo Time; RF, Radiofrequency; FOV, Field-of-View.
