# Supplementary figures and images for: High-Contrast Lumbar Spinal Bone Imaging Using a 3D Slab-Selective UTE Sequence
Source: Front Endocrinol (Lausanne). 2022 Jan 7;12:800398. doi: 10.3389/fendo.2021.800398 (PMC8777294; doi:10.3389/fendo.2021.800398)

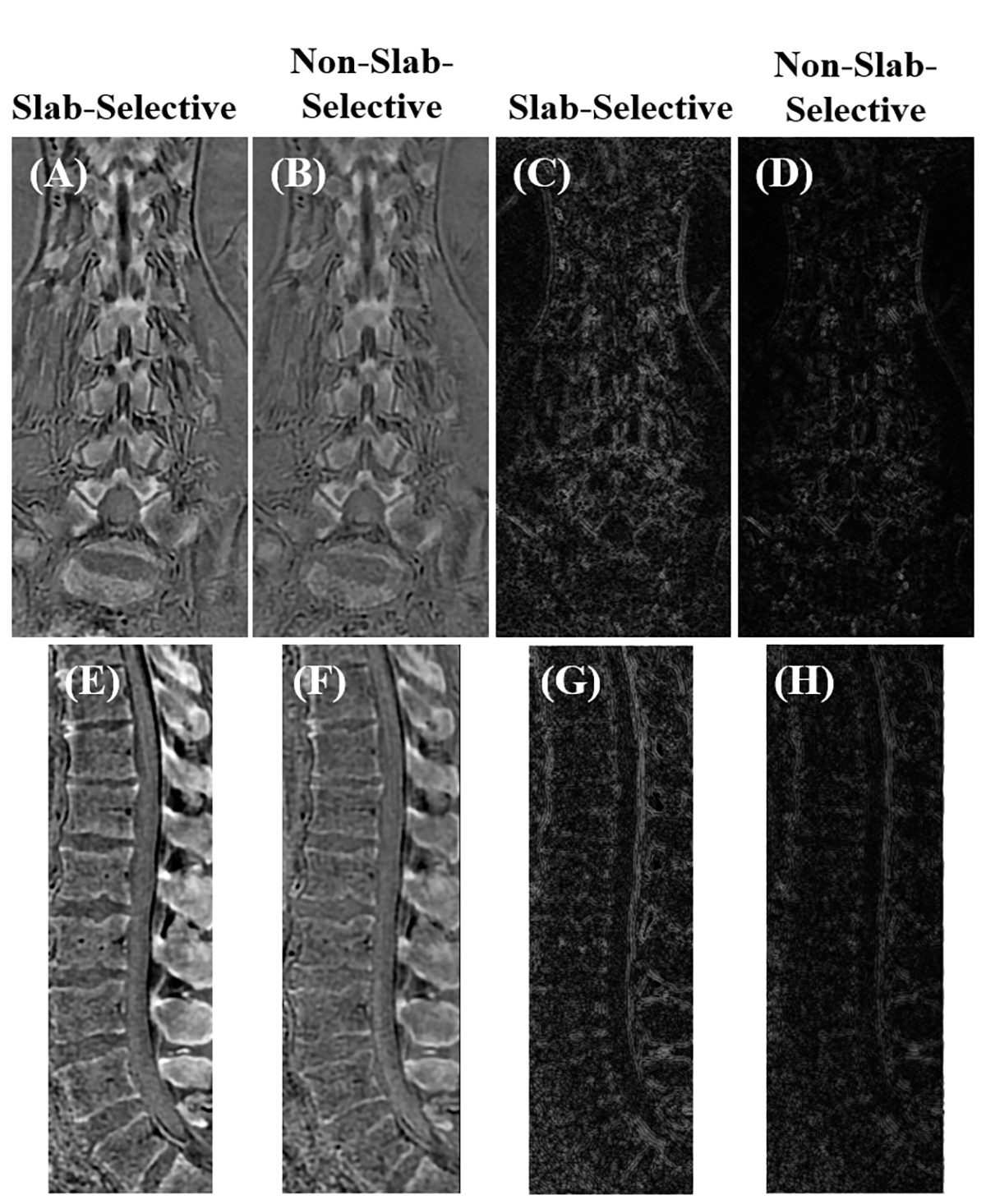

Supplement: Supplementary Figure 1 — Coronal (A–D) and sagittal (E–H) UTE images with slab-selective and non-slab-selective RF pulses for signal excitation. Greater bone sharpness was observed in the slab-selective UTE images (A, E) than the corresponding non-slab-selective images (B, F). The corresponding focus measure for the Laplacian filtered slab-selective UTE images (C, G) shows about 2.5x higher value than that of the non-slab-selective images (D, H). [file Image_1.tif]

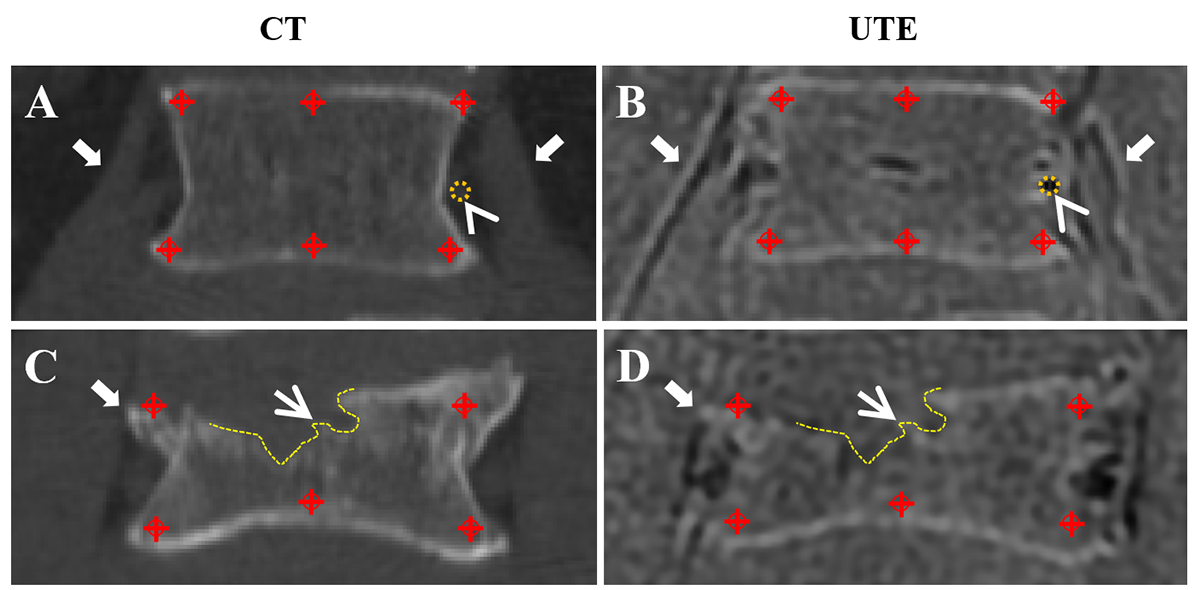

Supplement: Supplementary Figure 2 — Volumetric automatic rigid registration between CT and 3D UTE MRI on a normal vertebral body (A, C) and a fractured vertebral body (B, D). A centered initialization was followed by rigid registration. The red crosses represent corresponding points automatically detected by the registration algorithm. The yellow dashed lines (fracture) and orange dashed circles (vessel) have been drawn to highlight regions for visual comparison. [file Image_2.tif]

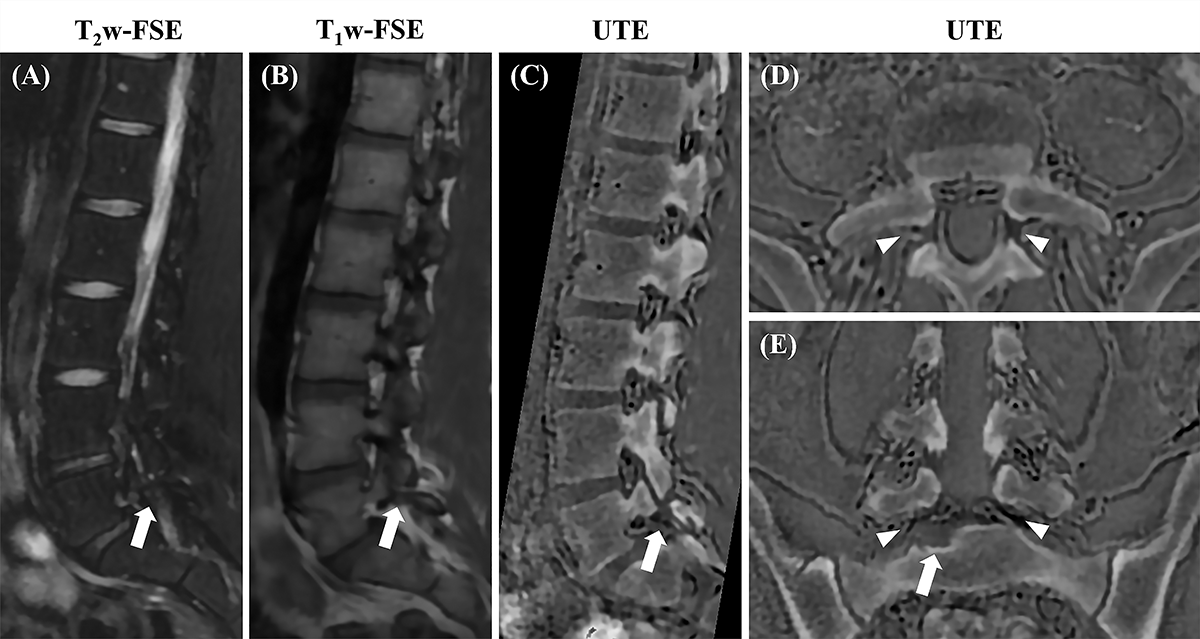

Supplement: Supplementary Figure 3 — Sagittal T2w-FS and T1w-FSE images (A, B) and sagittal (C), axial (D), and coronal (E) 3D slab-selective UTE images from a 35-year-old male patient with low back pain (T12-L5). A spondylolysis of the pars interarticularis of L5 with anterior spondylolisthesis of L5 is better distinguished on the 3D slab-selective UTE MR sequence [arrows in (A–C)]. An axial oblique reconstructed image of the UTE MR sequence shows the bilateral fractures of the pars interarticularis in detail [arrows in (D)]. The coronal reconstructed image of the 3D slab-selective UTE MR sequence shows the corresponding expected location of the fractures [arrowheads in (E)] as well as a Schmorl’s node in the superior endplate of S1 [arrow in (E)]. [file Image_3.tif]
